# Supplementary material for: Efficacy and safety of acupuncture for postpartum hypogalactia: A systematic review and meta-analysis of randomized controlled trials
Source: PLoS One. 2024 Jun 6;19(6):e0303948. doi: 10.1371/journal.pone.0303948 (PMC11156417; doi:10.1371/journal.pone.0303948)
Supplement: S1 Fig — (DOCX) [file pone.0303948.s001.docx]

**Supplementary Figure 1.** **Subgroup analyses for the outcomes of included studies**

**
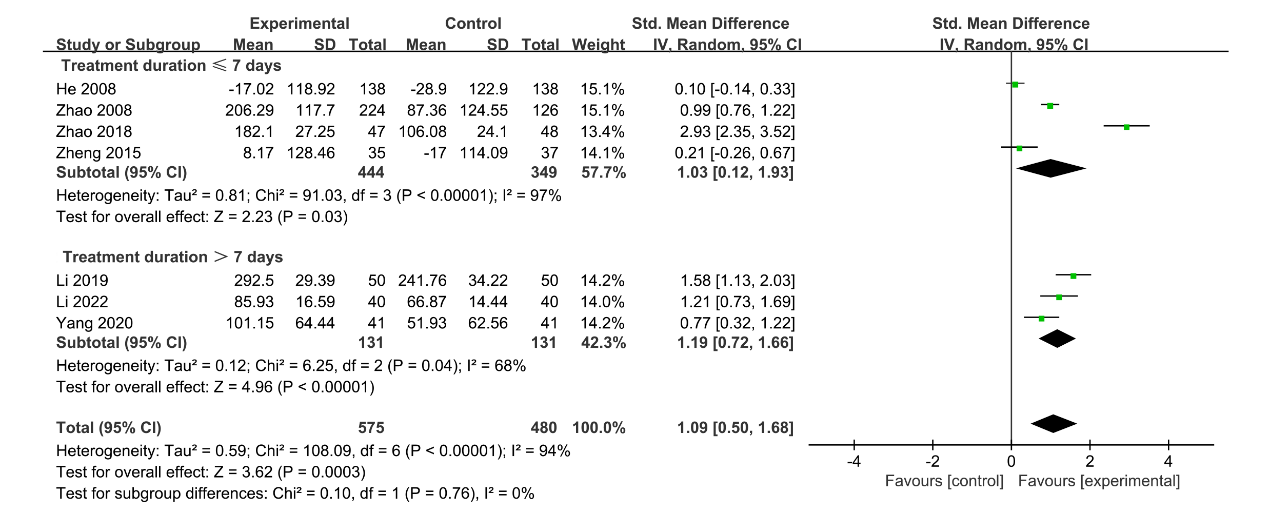
**

(A) Subgroup analysis of serum prolactin level according to different treatment durations

**
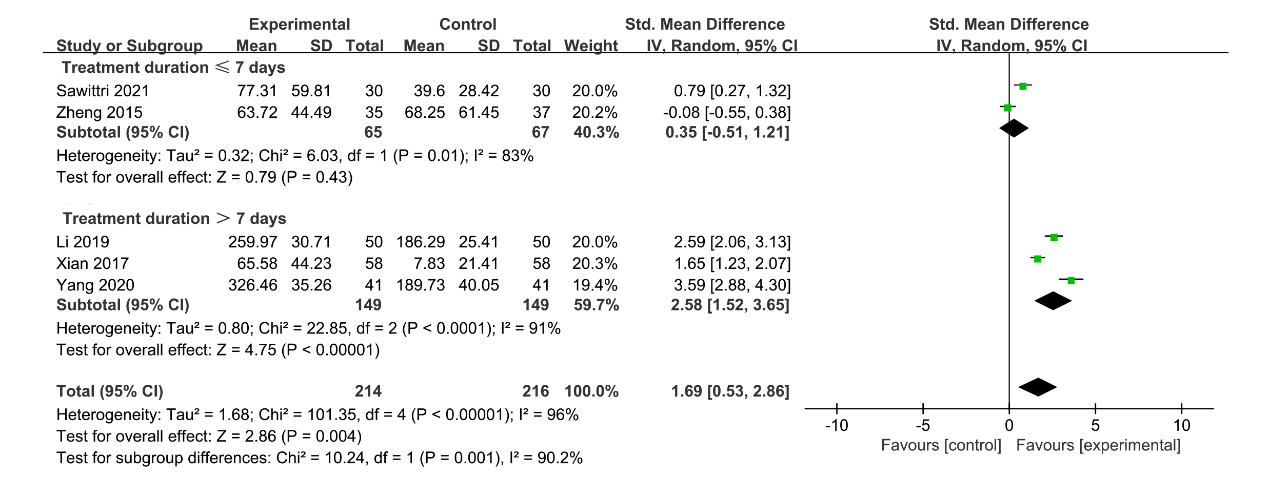
**

(B) Subgroup analysis of milk secretion volume according to different treatment durations

**
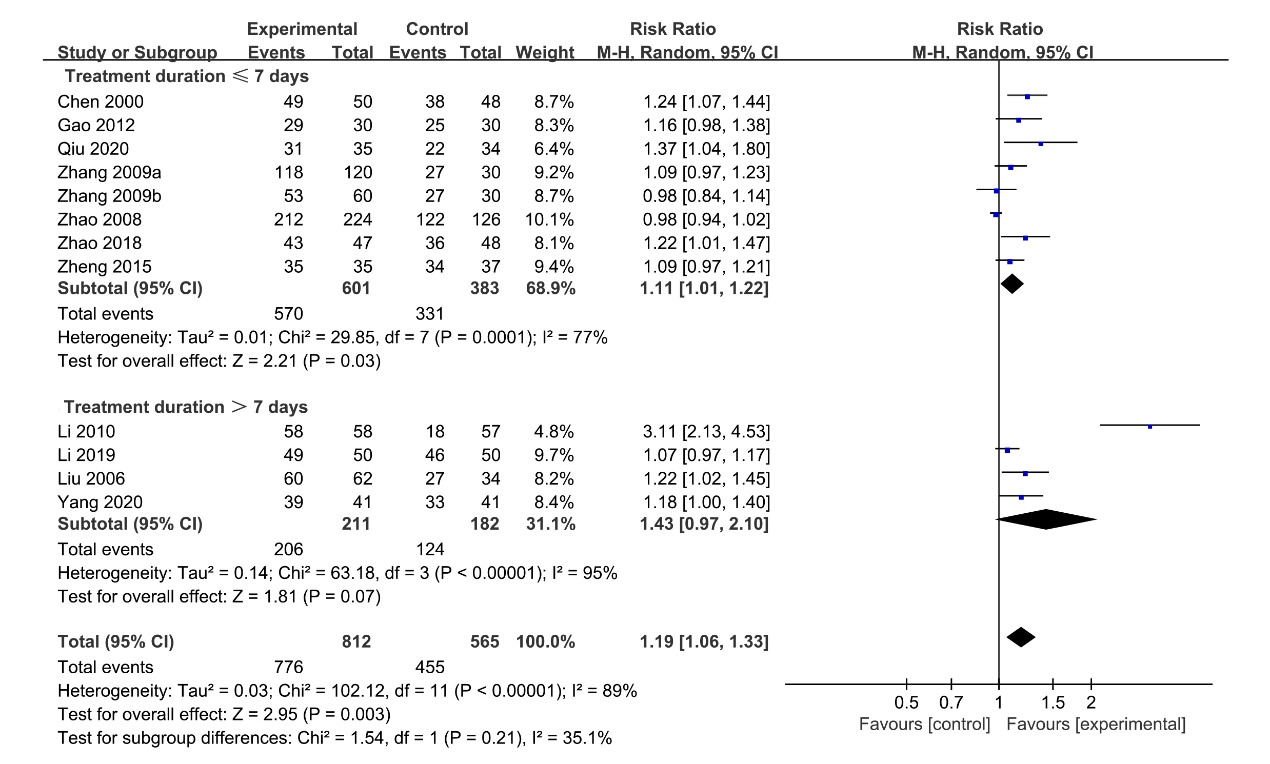
**

(C) Subgroup analysis of total effective rate according to different treatment durations

**
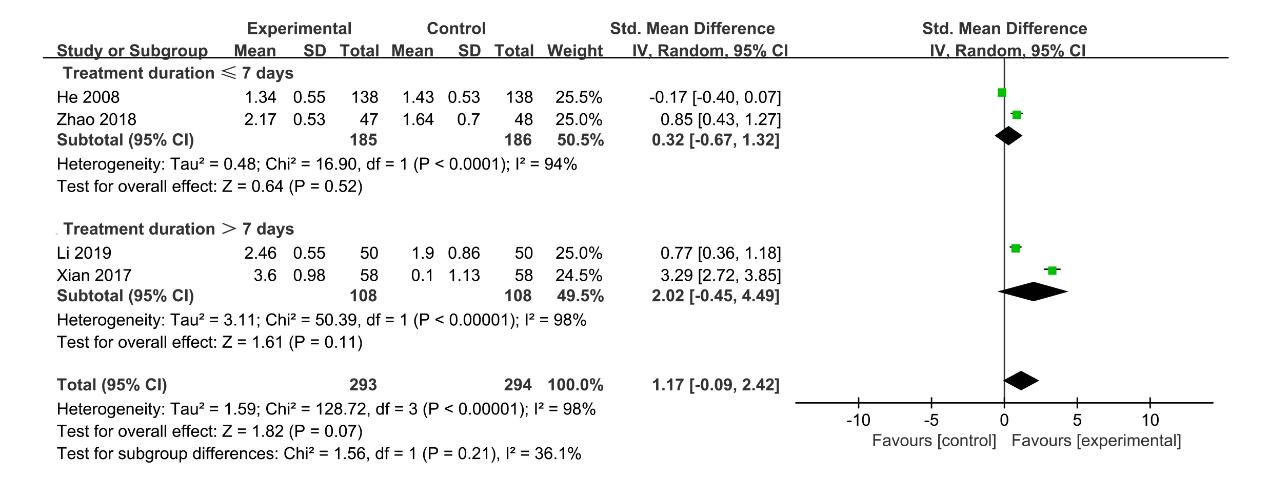
**

(D) Subgroup analysis of mammary fullness degree according to different treatment durations
