# Supplementary material for: Efficacy and safety of acupuncture for postpartum hypogalactia: A systematic review and meta-analysis of randomized controlled trials
Source: PLoS One. 2024 Jun 6;19(6):e0303948. doi: 10.1371/journal.pone.0303948 (PMC11156417; doi:10.1371/journal.pone.0303948)
Supplement: S2 Fig — (DOCX) [file pone.0303948.s002.docx]

**Supplementary Figure 2. Sensitivity analyses for different outcomes**

(A). Sensitivity analysis of acupuncture vs. Chinese herb for serum prolactin level

(B). Sensitivity analysis of acupuncture vs. Chinese herb for total effective rate

(C). Sensitivity analysis of acupuncture + Chinese herb vs. Chinese herb for total effective rate
