# Supplementary material for: Efficacy and safety of acupuncture for postpartum hypogalactia: A systematic review and meta-analysis of randomized controlled trials
Source: PLoS One. 2024 Jun 6;19(6):e0303948. doi: 10.1371/journal.pone.0303948 (PMC11156417; doi:10.1371/journal.pone.0303948)

**Supplementary Figure 3. Forest plot of total effective rate for acupuncture + Chinese herb vs. Chinese herb with Chen-2017 removed**


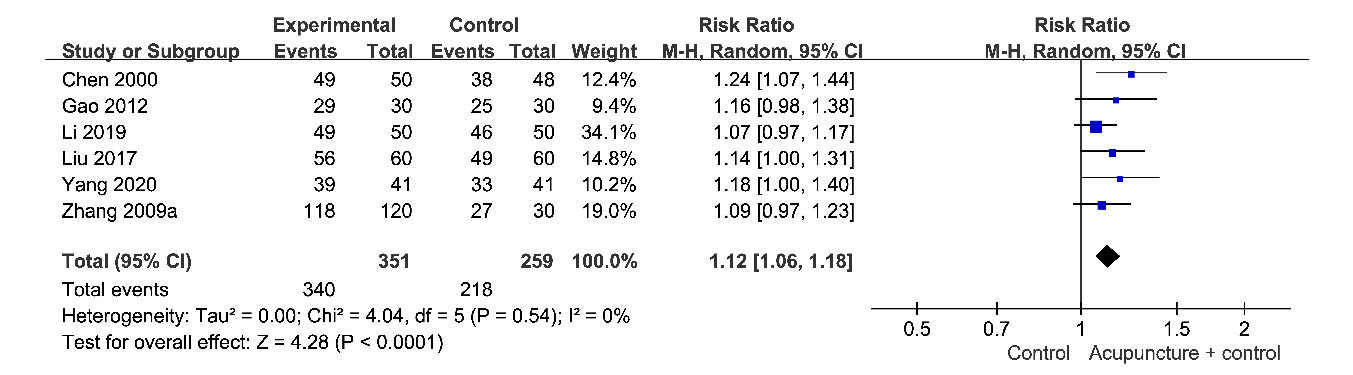

Supplement: S3 Fig — (DOCX) [file pone.0303948.s003.docx]
