# Supplementary material for: Efficacy and safety of acupuncture for postpartum hypogalactia: A systematic review and meta-analysis of randomized controlled trials
Source: PLoS One. 2024 Jun 6;19(6):e0303948. doi: 10.1371/journal.pone.0303948 (PMC11156417; doi:10.1371/journal.pone.0303948)
Supplement: S1 Table — (DOCX) [file pone.0303948.s004.docx]

**Supplementary Table 1. Full-text articles excluded with reasons**

| **Full-text articles excluded** | **Reasons** |
| --- | --- |
| Quan, L.Q. 2022 [1] | Ineligible intervention |
| Maged, A.M. 2020 [2] | Ineligible intervention |
| Li, L.P. 2015 [3] | Ineligible intervention |
| Yang, S.M. 2016 [4] | Ineligible intervention |
| Z. Z. Zhang, 2022 [5] | Ineligible comparator |
| Liu, P.S. 2022 [6] | Ineligible comparator |
| Hu, W.J. 2022 [7] | Ineligible comparator |
| Quan, L.Q. 2022 [8] | Ineligible comparator |
| Hu, M.J. 2022 [9] | Ineligible comparator |
| Yuan, Y. 2021 [10] | Ineligible comparator |
| Liu, M. F. 2021 [11] | Ineligible comparator |
| Li, S.N. 2020 [12] | Ineligible comparator |
| Chen, F. 2020 [13] | Ineligible comparator |
| Chen, M.F. 2018 [14] | Ineligible comparator |
| Peng, Y. 2016 [15] | Ineligible comparator |
| Nie, L.F. 2016 [16] | Ineligible comparator |
| Li, Z. 2016 [17] | Ineligible comparator |
| Lei, J.P. 2014 [18] | Ineligible comparator |
| Wang, Q.F. 2013 [19] | Ineligible comparator |
| Zhang, S.F. 2012 [20] | Ineligible comparator |
| Li, Z.T. 2006 [21] | Ineligible comparator |
| Bi, J.X. 2017 [22] | Ineligible comparator |
| Chen, X.L. 2018 [23] | Ineligible comparator |
| Li, K. 2000 [24] | Ineligible comparator |
| Sui, M.F. 2004 [25] | Ineligible comparator |
| He, X. 2015 [26] | Ineligible comparator |
| Li, J. H. 2016 [27] | Ineligible comparator |
| Li, J. H. 2016 [28] | Ineligible comparator |
| Li, Q. X. 2016 [29] | Ineligible comparator |
| Zhang, J.Y. 2016 [30] | Ineligible comparator |
| Gao, X. Y. 2017 [31] | Ineligible comparator |
| Yao, Y.Y. 2019 [32] | Ineligible comparator |
| Zhao, Y.Y. 2020 [33] | Ineligible comparator |
| R.B.R. z2z. 2020 [34] | Clinical trial registration |
| R.B.R. hhy. 2020 [35] | Clinical trial registration |
| Nct. 2020 [36] | Clinical trial registration |
| Actrn 2011 [37] | Clinical trial registration |
| Tctr. 2020 [38] | Clinical trial registration |
| Zhao, R. 2018 [39] | Duplicate content |
| Huang, T. 2007 [40] | Duplicate content |
| Huang, T. 2008 [41] | Duplicate content |
| Zhan, J. L. 2023 [42] | Non-RCT |
| Feng, X.Z. 2011 [43] | Non-RCT |
| Sun, J.S. 2008 [44] | Non-RCT |
| Mao, L.J. 2016 [45] | Non-RCT |
| Chen, R.Y. 2011 [46] | Non-RCT |
| Qin, X.L. 2010 [47] | Non-RCT |
| Sun, J.S. 2007 [48] | Non-RCT |
| Chen, H.H. 2009 [49] | Non-RCT |
| Wang, X.Y. 2007 [50] | Non-RCT |
| Zhao, Y. 2006 [51] | Non-RCT |
| Liu, Y.F. 1995 [52] | Non-RCT |
| Li, S.P. 1995 [53] | Non-RCT |
| Liang, S.Y. 1992 [54] | Non-RCT |
| Zhao, H.L. 2013 [55] | Non-RCT |
| Tureanu, L. 1994 [56] | Non-RCT |
| Zhu, P.D. 1958 [57] | Non-RCT |
| Zhao, Y. 2007 [58] | Non-RCT |
| Zhao, R. 2019 [59] | Non-RCT |
| Luo, X.Y. 2015 [60] | Ineligible participants |
| Chen, C. 2015 [61] | Ineligible participants |
| Marková, H. 1987 [62] | Full-text unavailable |
| Unknown 2000 [63] | Full-text unavailable |

**Reference:**

1. Quan LQ, Chen LZ, Fan ML, Meng LN. Clinical effect of demand-oriented nursing combined with midnight-midday ebb flow acupuncture in the treatment of postpartum hypogalactia. Zhong Wai Yi Liao. 2022;41(10):129-33. doi: 10.16662/j.cnki.1674-0742.2022.10.129

2. Maged AM, Hassanin ME, Kamal WM, Abbassy AH, Alalfy M, Askalani AN, et al. Effect of Low-Level Laser Therapy versus Electroacupuncture on Postnatal Scanty Milk Secretion: a Randomized Controlled Trial. American journal of perinatology. 2020;37(12):1243‐9. doi: 10.1055/s-0039-1693428

3. Li LP, Bao YH, Zhou M, Huang JX. Effect of catgut embedding intervention on postpartum lactation of puerperium women with Qi and Blood deficiency. Proceedings of the 11th National Academic Conference on External Treatment of Traditional Chinese Medicine2015. p. 348-51.

4. Yang SM, Sang WS, Liu JF, He XJ, Zhang YF, Zhai XH. Effect of breast stimulation combined with dietary therapy on breastfeeding of premature infants and maternal and child health. Zhong Guo Fu You Bao Jian. 2016;31(16):3273‐5. doi: 10.7620/zgfybj.j.issn.1001-4411.2016.16.31

5. Zhang ZZ, Hua ZZ, Wei XX, Hu CF, Wu AQ. Effect of meridian flow acupuncture combined with acupoint selection based on syndrome differentiation on postpartum breast filling and lactation. Medical Journal of West China. 2022;34(10):1542-6.

6. Liu PS, Tan GX. Clinical Study of Acupuncture Combined with MuguaJiyudecoration in the Treament of Postpartum Hypogalactia Due to Deficiency of Qi and Blood. Chinese traditional medicine modern distance education. 2022;20(20):122-4.

7. Hu WJ, Sun AJ, Li J, Lu Y, Xin ZY, Tao CY. Clinical observation of acupuncture combined with auricular plaster therapy in treating postpartum lactation with syndrome of qi and blood deficiency. China's Naturopathy. 2023;31(02):50-2. 10.19621/j.cnki.11-3555/r.2023.0216

8. Quan LQ, Chen LZ, Fan ML, Meng LN, 全柳青, 陈莲珠, et al. Clinical Effect of Demand-oriented Nursing Combined with Meridian Injection in Patients with Postpartum Hypogalactia. China Foreign Medical Treatment. 2022;41(10):129-33. 10.16662/j.cnki.1674-0742.2022.10.129

9. Hu MJ. Influence of Acupuncture and Medicine Combined with Traditional Chinese Medicine Characteristic Intervention with Deficiency of Qi and Blood. Guang Ming Zhong Yi. 2022;37(15):2762-4.

10. Yuan Y, Kang LD, Hou Y. Effect of acupuncture and medicine combined with traditional Chinese medicine emotional nursing on postpartum breastfeeding and prolactin level of puerpera with qi and blood deficiency. Journal of Changchun University of Chinese Medicine. 2021;37(2):416-9. doi: 10.13463/j.cnki.cczyy.2021.02.048

11. Liu MF, Bi XX, Geng DD, Jia XJ. Observation on Clinical Effect and Safety of Meridional Flow Injection Combined with Syndrome Differentiation Acupuncture in Treatment of Postpartum Lactation. Zhonghua Zhong Yi Yao Xue Kan. 2021;39(06):245-8. doi: 10.13193/j.issn.1673-7717.2021.06.056

12. Li SN. Clinical observation on the treatment of postpartum hypogalactia with acupuncture and medicine combined with massage. Zhong Yi Lin Chuang Yan Jiu. 2020;12(21):118-20. doi: 10.3969/j.issn.1674-7860.2020.21.046

13. Chen F. Clinical effect of acupuncture combined with modified Tongru decoction in the treatment of postpartum hypogalactia. Zhong Guo Dang Dai Yi Yao. 2020;27(18):159-62.

14. Chen MF, Xie JJ. Clinical study on 46 cases of postpartum hypogalactia treated by electroacupuncture at Danzhong point combined with computer medium frequency (diothermy) therapeutic apparatus. He Bei Zhong Yi. 2018;40(03):374-7. doi: 10.3969/j.issn.1002-2619.2018.03.012

15. Peng Y. Effect of traditional Chinese medicine combined with warming acupuncture on postpartum hypogalactia. Yi Liao Zhuang Bei. 2016;29(24):66-7.

16. Nie LF. Acupuncture and tuina in the treatment of 60 cases of postpartum hypogalactia. Xin Jiang Zhong Yi Yao. 2016;34(2):29-30.

17. Li Z, Chen YC, Su LQ. Treatment of postpartum lactation by acupuncture with Zang time phase regulation. Journal of Changchun University of Chinese Medicine. 2016;32(1):139-41. doi: 10.13463/j.cnki.cczyy.2016.01.047

18. Lei JP. Clinical effect of acupuncture combined with local massage on postpartum hypogalactia. Zhong Guo Yi Yao Zhi Nan. 2014;12(07):219-20. doi: 10.15912/j.cnki.gocm.2014.07.009

19. Wang QF. Effect of acupuncture and tuina combined with cupping in the treatment of postpartum hypogalactia. Zhong Guo Liao Yang Yi Xue. 2013;22(3):246-7. doi: 10.13517/j.cnki.ccm.2013.03.016

20. Zhang SF. Clinical study on the treatment of postpartum hypogalacia of liver depression and qi stagnation by semi-acupuncture combined with massage. Thesis, Hebei Medical University; 2012.

21. Li ZT. 55 cases of postpartum hypogalactia were treated by acupuncture. Shan Xi Zhong Yi. 2006;27(2):226-7. doi: 10.3969/j.issn.1000-7369.2006.02.062

22. Bi JX, Du M, R., Liu Y, Sun CX. Clinical study on the combination of acupuncture and medication in the prevention of hypogalactia after cesarean section. Yin Shi Bao Jian. 2017;4:89-90.

23. Chen XL, Tian PX, Liu XH, Chen LM. Clinical effect of acupuncture combined with massage in the treatment of postpartum hypogalactia: Xingtai County Hospital; 2018 2018-01-18.

24. Li K. A Pilot Study to Evaluate the Effect of Acupuncture on Increasing Milk Supply of Lactating Mothers. Thesis, Victoria University of Technology; 2000.

25. Sui MF, Zhao Q. Clinical observation of acupuncture promoting postpartum milk secretion. Journal of Clinical Acupuncture and Moxibustion. 2004;(12):31. doi: 10.3969/j.issn.1005-0779.2004.12.017

26. He X, Xu YQ, D J. Clinical efficacy of postpartum acupuncture combined with massage in the treatment of hypogalactia of liver depression and qi stagnation. Zhong Wai Nv Xing Jian Kang Yan Jiu. 2015;8:229-30.

27. Li JH, editor Clinical effect of acupuncture combined with local massage in the treatment of postpartum hypogalactia of Qi and blood deficiency. The inaugural meeting of the Digital Traditional Chinese Medicine Branch of the International Digital Medicine Association and the first Academic Exchange meeting of Digital Traditional Chinese Medicine; 2016.

28. Li JH, Li QX. Clinical observation on 100 cases of postpartum hypogalactia with Qi and blood deficiency treated by acupuncture. Zhong Guo Fu You Bao Jian Yan Jiu. 2016;27(S2):327.

29. Li QX, Li JH. Clinical study of acupuncture in the treatment of postpartum hypogalactia of Qi and blood deficiency. Journal of Clinical Acupuncture and Moxibustion. 2016;32(12):28-30.

30. Zhang JY, Cui M, Yao W, Li CX, Ye SQ, Xu YP. Clinical effect of Tongrou decoction combined with acupuncture in the treatment of 100 cases of postpartum hypogalactia. Zhejiang Journal of Traditional Chinese Medicine. 2016;51(08):567-8. doi: 10.13633/j.cnki.zjtcm.2016.08.015

31. Gao XY. Study on the effect of acupuncture and tuina on the lactation time of primipara. Mu Ying Shi Jie. 2017;(20):63. doi: 10.3969/j.issn.1671-2242.2017.20.059

32. Yao YY, Guo XQ, Han WN, Zeng L. Clinical observation on 35 cases of postpartum hypogalactia with phlegm turbidity blocking collateral type treated by acupuncture combined with acupoint massage. Jiangsu Zhong Yi Yao. 2019;51(11):60-2.

33. Zhao YY. Observation effect of Acupuncture Combined with Plum Blossom Acupuncture on Postpartum Milk Deficiency. Yi Xue Xin Xi. 2020;33(S01):26-8. doi: 10.3969/j.issn.1006-1959.2020.z1.017

34. z2z RBR. Use of ear acupuncture to help maintain breastfeeding until 6 months of age. <https://trialsearchwhoint/Trial2aspx?TrialID=RBR-8z2z82>. 2020;

35. hhy RBR. Effect of auricular acupuncture in breastfeeding. <https://trialsearchwhoint/Trial2aspx?TrialID=RBR-563hhy>. 2020;

36. Nct. Effectiveness of Press Tack Needle Acupuncture in Treating Lactation Insufficiency. <https://clinicaltrialsgov/show/NCT04416880>. 2020;

37. Actrn. Ear acupuncture for anxiety in lactating mothers: a randomized controlled trial. <http://wwwwhoint/trialsearch/Trial2aspx?TrialID=ACTRN12611000025932>. 2011;

38. Tctr. Acupuncture to boost breast milk in postpartum hypogalactia : randomized controlled trial. <https://trialsearchwhoint/Trial2aspx?TrialID=TCTR20200306003>. 2020;

39. Zhao R, Wang XX, Li YE, Ma ZX. Clinical observation of acupuncture plus electroacupuncture in the treatment of postpartum hypogalactia. Shanghai Journal of Acupuncture and Moxibustion. 2018;37(10):1160-4. doi: 10.13460/j.issn.1005-0957.2018.10.1160

40. Huang T, Chen BY, He JQ, Bai J, Gu M, Cao H-t, et al. Clinical effect of acupuncture at Danzhong on postpartum hypogalactia. Shanghai Journal of Acupuncture and Moxibustion. 2007;(11):3-5. doi: 10.13460/j.issn.1005-0957.2007.11.002

41. Huang T, Chen BY, He JQ, Bai J, Gu M, Cao H-t, et al. Study on clinical efficacy of needling Danzhong(CV 17) in treating postpartum hypogalactia. Journal of Acupuncture and Tuina Science. 2008;6(1):27-31. doi: 10.1007/s11726-008-0027-y

42. Zhan J, Huang N, Meng N, Qiu Y, Zhu Y, Xu Y, et al. Lactation prescription plus acupoint stimulation improves breastfeeding quality and alleviates breast tenderness in parturients undergoing cesarean section. 2023;(1943-8141 (Print))

43. Feng XZ. Clinical study of acupuncture combined with medium frequency electroacupuncture in the treatment of postpartum hypogalactia in Xining area: Qinghai Red Cross Hospital; 2011 2011-07-26.

44. Sun JS, editor Clinical observation on 30 cases of postpartum hypogalactia treated by acupuncture. The 11th National Symposium on Auricular Diagnosis and Treatment, Forum on Contemporary Clinical Treatment and Symposium on Ten Central and Western Provinces; 2008.

45. Mao LJ. Clinical effect of acupuncture combined with Zengru decoction in the treatment of hypogalactia of Qi and Blood deficiency. Zhong Guo Yao Wu Jing Ji Xue. 2016;11(01):76-8. doi: 10.12010/j.issn.1673-5846.2016.01.038

46. Chen RY. Clinical observation on 40 cases of postpartum hypogalactia treated by acupuncture and medicine. He Bei Zhong Yi. 2011;33(02):248-9. doi: 10.3969/j.issn.1002-2619.2011.02.053

47. Qin XL, Teng H, Xiao DM, Xie Y. Clinical observation on 56 cases of postpartum hypogalacia treated by letting blood of acupuncture at Danzhong and Shaoze Guang Ming Zhong Yi. 2010;25(08):1456-7. doi: 10.3969/j.issn.1003-8914.2010.08.084

48. Sun JS, editor Clinical observation on 30 cases of postpartum hypogalactia treated by acupuncture. The 15th National Acupuncture-moxibustion Symposium of Clinical Branch of Chinese Acupuncture-Moxibustion Society; 2007.

49. Chen HH. Theory and clinical study of integrated traditional Chinese and western medicine intervention for postpartum hypogalactia. Thesis, China Academy of Chinese Medical Science; 2009.

50. Wang XY. Clinical experience of acupuncture combined with massage in the treatment of postpartum hypogalactia. Ke Ji Xin Xi. 2007;238(26):257.

51. Zhao Y, Guo H. The therapeutic effects of acupuncture in 30 cases of postpartum hypogalactia. Journal of Traditional Chinese Medicine. 2006;26(1):29-30.

52. Liu YF. Clinical evaluation of acupuncture and moxibustion in the treatment of lactation insufficiency. Zhong Guo Zhong Yi Yao Xin Xi Za Zhi. 1995;(06):9-10.

53. Li SP, Bai SF. Clinical effect of acupuncture in the treatment of insufficient milk secretion. Guo Wai Yi Xue. 1995;(01):48.

54. Liang SY, Wang JH. Clinical observation of 50 cases of hypogalactia treated by acupuncture. Journal of Gansu College of Traditional Chinese Medicine 1992;(02):26.

55. Zhao HL, Wang JY, Li HX, Li L. Clinical observation of acupuncture combined with local massage in the treatment of postpartum hypogalactia: The third staff Hospital of Baotou Steel Group; 2013 2013-12-23.

56. Tureanu L, Tureanu V. A clinical evaluation of the effectiveness of acupuncture for insufficient lactation. American Journal of Acupuncture. 1994;22(1):23-7.

57. Zhu PD, Yan RY. Clinical observation and research of acupuncture and moxibustion in weaning. Chinese Journal of Obstetrics and Gynecology. 1958;(04):321-7.

58. Zhao Y, Li Q, Ding QL, Wang YP, Huang CL. clinical effect of acupuncture on endocrinology in patients with postpartum hypogalactia: Shijiazhuang Municipal Hospital of Traditional Chinese Medicine; 2007 2007-10-23.

59. Zhao R, Sun YJ, Cui JM, Qiao LJ, Zhan M. Clinical observation on the treatment of postpartum hypogalactia by acupuncture and electroacupuncture: Baoding First Hospital of Traditional Chinese Medicine; 2019 2019-02-28.

60. Luo XY, Luo Y. Effect of holographic acupuncture analgesia on lactation after cesarean section. Proceedings of 2015 clinical critical care experience Exchange Summit Forum2015. p. 739.

61. Chen C. Study on the optimized treatment of traditional Chinese medicine for postpartum conditioning and promoting postpartum recovery. Zhong Yi Lin Chuang Yan Jiu. 2015;7(15):90-1,3.

62. Marková H, Marek J, Joza V. The effect of acupuncture on lactation. Ceskoslovenská gynekologie. 1987;52(10):783-6.

63. ACUPUNCTURE TRIAL TO STUDY BREASTFEEDING PROBLEMS. Australian Nursing Journal. 2000;8(2):37.
