# Supplementary material for: Efficacy and safety of acupuncture for postpartum hypogalactia: A systematic review and meta-analysis of randomized controlled trials
Source: PLoS One. 2024 Jun 6;19(6):e0303948. doi: 10.1371/journal.pone.0303948 (PMC11156417; doi:10.1371/journal.pone.0303948)
Supplement: S2 Table — (DOCX) [file pone.0303948.s005.docx]

**Supplementary Table 2. Egger’s test** **for different outcomes**

| **Outcomes** | **Number of studies** | **Egger's test** |
| --- | --- | --- |
| Serum PRL level | 7 | 0.186 |
| MSV | 5 | 0.269 |
| TER | 13 | 0.000 |
| MFD | 4 | 0.065 |
| EBR | 2 | Not applicable |

*Notes.* PRL: prolactin; MSV: milk secretion volume; TER: total effective rate; MFD: mammary fullness degree; EBR: exclusive breastfeeding rate.
