# Supplementary material for: Efficacy and safety of acupuncture for postpartum hypogalactia: A systematic review and meta-analysis of randomized controlled trials
Source: PLoS One. 2024 Jun 6;19(6):e0303948. doi: 10.1371/journal.pone.0303948 (PMC11156417; doi:10.1371/journal.pone.0303948)
Supplement: S1 File — (DOCX) [file pone.0303948.s006.docx]

**Supplementary File 2. PRISMA checklist**

| **Section and Topic** | **Item #** | **Checklist item** | **Location where item is reported** |
| --- | --- | --- | --- |
| **TITLE** | | |  |
| Title | 1 | This study is identified as a meta-analysis | 1 |
| **ABSTRACT** | | |  |
| Abstract | 2 | This summary includes the Background, methods, results and discussion | 2-3 |
| **INTRODUCTION** | | |  |
| Rationale | 3 | Described in the introduction | 4-5 |
| Objectives | 4 | Stated in the introduction | 6 |
| **METHODS** | | |  |
| Eligibility criteria | 5 | This paper provided a detailed description of the inclusion and exclusion criteria | 7-8 |
| Information sources | 6 | Specify all databases, registers, websites, organisations, reference lists and other sources searched or consulted to identify studies. Specify the date when each source was last searched or consulted. | 6-7 |
| Search strategy | 7 | Present the full search strategies for all databases, registers and websites, including any filters and limits used. | 7 |
| Selection process | 8 | The screening process is included in the data collection | 7 |
| Data collection process | 9 | We describe the data collection in detail | 8-9 |
| Data items | 10a | We provide a detailed description of the outcome indicators | 8 |
|  | 10b | List and define all other variables for which data were sought (e.g. participant and intervention characteristics, funding sources). Describe any assumptions made about any missing or unclear information. | 9 |
| Study risk of bias assessment | 11 | We provide a detailed description of the risk assessment tool | 9 |
| Effect measures | 12 | In the methodology section, we made a specific introduction to the measurement of each outcome indicator | 9-10 |
| Synthesis methods | 13a | Describe the processes used to decide which studies were eligible for each synthesis (e.g. tabulating the study intervention characteristics and comparing against the planned groups for each synthesis (item #5)). | 9-10 |
|  | 13b | Describe any methods required to prepare the data for presentation or synthesis, such as handling of missing summary statistics, or data conversions. | 9-10 |
|  | 13c | Describe any methods used to tabulate or visually display results of individual studies and syntheses. | 9-10 |
|  | 13d | Describe any methods used to synthesize results and provide a rationale for the choice(s). If meta-analysis was performed, describe the model(s), method(s) to identify the presence and extent of statistical heterogeneity, and software package(s) used. | 10 |
|  | 13e | Describe any methods used to explore possible causes of heterogeneity among study results (e.g. subgroup analysis, meta-regression). | 10 |
|  | 13f | Describe any sensitivity analyses conducted to assess robustness of the synthesized results. | 10 |
| Reporting bias assessment | 14 | We introduced the method of measuring bias. | 10 |
| Certainty assessment | 15 | We used GRADE to evaluate the evidence for an outcome. | 10 |
| **RESULTS** | | |  |
| Study selection | 16a | Describe the results of the search and selection process, from the number of records identified in the search to the number of studies included in the review, ideally using a flow diagram. | 10-11 |
|  | 16b | Cite studies that might appear to meet the inclusion criteria, but which were excluded, and explain why they were excluded. | 11 |
| Study characteristics | 17 | Cite each included study and present its characteristics. | 11-15 |
| Risk of bias in studies | 18 | Present assessments of risk of bias for each included study. | 19 |
| Results of individual studies | 19 | For all outcomes, present, for each study: (a) summary statistics for each group (where appropriate) and (b) an effect estimate and its precision (e.g. confidence/credible interval), ideally using structured tables or plots. | 20-24 |
| Results of syntheses | 20a | For each synthesis, briefly summarise the characteristics and risk of bias among contributing studies. | 20-24 |
|  | 20b | Present results of all statistical syntheses conducted. If meta-analysis was done, present for each the summary estimate and its precision (e.g. confidence/credible interval) and measures of statistical heterogeneity. If comparing groups, describe the direction of the effect. | 20-24 |
|  | 20c | We conducted subgroup analysis to further study | 20-24 |
|  | 20d | Present results of all sensitivity analyses conducted to assess the robustness of the synthesized results. | 27 |
| Reporting biases | 21 | Present assessments of risk of bias due to missing results (arising from reporting biases) for each synthesis assessed. | 27 |
| Certainty of evidence | 22 | We used GRADE to evaluate the evidence for an outcome. | 28 |
| **DISCUSSION** | | |  |
| Discussion | 23a | Provide a general interpretation of the results in the context of other evidence. | 30-31 |
|  | 23b | Discuss any limitations of the evidence included in the review. | 35 |
|  | 23c | Discuss any limitations of the review processes used. | 35 |
|  | 23d | Discuss implications of the results for practice, policy, and future research. | 33-34 |
| **OTHER INFORMATION** | | |  |
| Registration and protocol | 24a | The registration information is the same as that in this document. Registration No.: CRD42022351849 | 6 |
|  | 24b | Indicate where the review protocol can be accessed, or state that a protocol was not prepared. | 6 |
|  | 24c | The registration information can be seen on prospero | 6 |
| Support | 25 | Describe sources of financial or non-financial support for the review, and the role of the funders or sponsors in the review. | - |
| Competing interests | 26 | No conflict of interest between authors | - |
| Availability of data, code and other materials | 27 | All data are published in the public database | - |

**Reference:**
