# Supplementary material for: Efficacy and safety of acupuncture for postpartum hypogalactia: A systematic review and meta-analysis of randomized controlled trials
Source: PLoS One. 2024 Jun 6;19(6):e0303948. doi: 10.1371/journal.pone.0303948 (PMC11156417; doi:10.1371/journal.pone.0303948)
Supplement: S2 File — (DOCX) [file pone.0303948.s007.docx]

**Supplementary File 1. Search strategies of each database**

**PubMed**

#1 "Acupuncture"[MeSH Terms] OR "Electroacupuncture"[MeSH Terms] OR "Acupuncture Therapy"[MeSH Terms] OR "Acupuncture Points"[MeSH Terms]

#2 "*Acupuncture"[Title/Abstract] OR "electroacupuncture"[Title/Abstract] OR "acupoint*"[Title/Abstract] OR "auriculotherapy"[Title/Abstract]

#3 #1 OR #2

#4 "Lactation"[MeSH Terms] OR "Lactation Disorders"[MeSH Terms] OR "Milk, Human"[MeSH Terms] OR "Milk Ejection"[MeSH Terms] OR "Breast Feeding"[MeSH Terms] OR "Postpartum Period"[MeSH Terms]

#5 "lactation"[Title/Abstract] OR "breast feeding"[Title/Abstract] OR "breastfeeding"[Title/Abstract] OR "hypogalactia"[Title/Abstract] OR "galactosis"[Title/Abstract] OR "colostrum"[Title/Abstract] OR "milk"[Title/Abstract] OR "postpartum"[Title/Abstract] OR "postnatal"[Title/Abstract] OR "puerperal"[Title/Abstract]

#6 #4 OR #5

#7 "Randomized Controlled Trials as Topic"[MeSH Terms] OR "random allocation"[MeSH Terms] OR "randomized controlled trial"[Publication Type] OR "controlled clinical trial"[Publication Type] OR "clinical trial"[Publication Type] OR "clinical study"[Title/Abstract] OR "trial"[Title/Abstract] OR "placebo"[Title/Abstract] OR "random*"[Title/Abstract]

#8 #3 AND #6 AND #7

**Web of Science**

TS=(‘*acupuncture’ OR ‘electroacupuncture’ OR ‘acupoint*’ OR ‘auriculotherapy’) AND TS=(‘lactation’ OR ‘breast feeding’ OR ‘breastfeeding’ OR ‘hypogalactia’ OR ‘galactosis’ OR ‘colostrum’ OR ‘milk’ OR ‘postpartum’ OR ‘postnatal’ OR ‘puerperal’) AND TS=(‘clinical study’ OR ‘trial’ OR ‘placebo’ OR ‘random*’)

**Cochrane Library**

#1 Mesh descriptor: [acupuncture] explode all trees

#2 Mesh descriptor: [electroacupuncture] explode all trees

#3 Mesh descriptor: [acupuncture therapy] explode all trees

#4 Mesh descriptor: [acupuncture points] explode all trees

#5 *acupuncture:ti,ab,kw OR electroacupuncture:ti,ab,kw OR acupoint*:ti,ab,kw OR "dry needle":ti,ab,kw OR auriculotherapy:ti,ab,kw

#6 #1 OR #2 OR #3 OR #4 OR #5

#7 Mesh descriptor: [Lactation] explode all trees

#8 Mesh descriptor: [Lactation Disorders] explode all trees

#9 Mesh descriptor: [Milk, Human] explode all trees

#10 Mesh descriptor: [Milk Ejection] explode all trees

#11 Mesh descriptor: [Breast Feeding] explode all trees

#12 Mesh descriptor: [Postpartum Period] explode all trees

#13 lactation:ti,ab,kw OR "breast feeding":ti,ab,kw OR breastfeeding:ti,ab,kw OR hypogalactia:ti,ab,kw OR galactosis:ti,ab,kw OR colostrum:ti,ab,kw OR milk:ti,ab,kw OR postpartum:ti,ab,kw OR postnatal:ti,ab,kw OR puerperal:ti,ab,kw

#14 #7 OR #8 OR #9 OR #10 OR #11 OR #12 OR #13

#15 Mesh descriptor: [Randomized Controlled Trials as Topic] explode all trees

#16 Mesh descriptor: [random allocation] explode all trees

#17 "clinical study":ti,ab,kw OR trial:ti,ab,kw OR placebo:ti,ab,kw OR random*:ti,ab,kw

#18 #15 OR #16 OR #17

#19 #6 AND #14 AND #18

**EMBASE**

#1 ‘Acupuncture’/exp/mj OR ‘Electroacupuncture’/exp/mj OR ‘Acupuncture Therapy’/exp/mj OR ‘Acupuncture Points’/exp/mj OR ‘acupuncture’:ti,ab,kw OR ‘electroacupuncture’:ti,ab,kw OR ‘acupoint*’:ti,ab,kw OR ‘auriculotherapy’:ti,ab,kw

#2 ‘Lactation’/exp/mj OR ‘Lactation Disorders’/exp/mj OR ‘Milk, Human’/exp/mj OR ‘Milk Ejection’/exp/mj OR ‘Breast Feeding’/exp/mj OR ‘Postpartum Period’/exp/mj OR ‘lactation’:ti,ab,kw OR ‘breast feeding’:ti,ab,kw OR ‘breastfeeding’:ti,ab,kw OR ‘hypogalactia’:ti,ab,kw OR ‘galactosis’:ti,ab,kw OR ‘colostrum’:ti,ab,kw OR ‘milk’:ti,ab,kw OR ‘postpartum’:ti,ab,kw OR ‘postnatal’:ti,ab,kw OR ‘puerperal’:ti,ab,kw

#3 ‘Randomized Controlled Trials as Topic’/exp/mj OR ‘random allocation’/exp/mj OR ‘clinical study’:ti,ab,kw OR ‘trial’:ti,ab,kw OR ‘placebo’:ti,ab,kw OR ‘random*’:ti,ab,kw

#4 #1 AND #2 AND #3

**EBSCO**

S1 SU ( "acupuncture" OR "electroacupuncture" OR "acupuncture therapy" OR "acupuncture points" ) OR TI ( "*acupuncture" OR "electroacupuncture" OR "acupoint" OR "dry needle" OR "auriculotherapy" ) OR AB ( "*acupuncture" OR "electroacupuncture" OR "acupoint" OR "dry needle" OR "auriculotherapy" )

S2 SU ( "lactation" OR "lactation disorders" OR "milk, human" OR "milk ejection" OR "breast feeding" OR "postpartum period" ) OR TI ( "lactation" OR "breast feeding" OR "breastfeeding" OR "hypogalactia" OR "galactosis" OR "colostrum" OR "milk" OR "postpartum" OR "postnatal" OR "puerperal" ) OR AB ( "lactation" OR "breast feeding" OR "breastfeeding" OR "hypogalactia" OR "galactosis" OR "colostrum" OR "milk" OR "postpartum" OR "postnatal" OR "puerperal" )

S3 SU ( "randomized controlled trials as topic" OR "random allocation" ) OR TI ( "clinical study" OR "trial" OR "placebo" OR "random*" ) OR AB ( "clinical study" OR "trial" OR "placebo" OR "random*" )

S4 S1 AND S2 AND S3

**Scopus**

#1 TITLE-ABS-KEY(*acupuncture OR electroacupuncture OR acupoint* OR auriculotherapy)

#2 TITLE-ABS-KEY(lactation OR "breast feeding" OR breastfeeding OR hypogalactia OR galactosis OR colostrum OR milk OR postpartum OR postnatal OR puerperal)

#3 TITLE-ABS-KEY("clinical study" OR trial OR placebo OR random*)

#4 #1 AND #2 AND #3

**China National Knowledge Infrastructure**

(TKA=(‘针刺’+‘针灸’+‘体针’+‘手针’+‘电针’+‘头针’+‘耳针’+‘腹针’+‘干针’) OR SU=(‘针刺疗法’+‘针刺’+‘针刺穴位’)) AND (TKA=(‘缺乳’+‘乳少’+‘少乳’+‘乳汁’+‘泌乳’+‘母乳’+‘哺乳’) OR SU=(‘泌乳障碍’+‘母乳喂养’)) AND (TKA=(‘随机’+‘临床研究’+‘临床试验’+‘RCT’) OR SU=(‘随机对照试验’+‘临床试验’))

**Chinese Biomedical Literature Database**

1 "针刺疗法" [加权:扩展] OR "针刺"[加权:扩展] OR "针刺穴位"[加权:扩展]

2 "针刺"[常用字段:智能] OR "针灸"[常用字段:智能] OR "体针"[常用字段:智能] OR "手针"[常用字段:智能] OR "电针"[常用字段:智能] OR "头针"[常用字段:智能] OR "耳针"[常用字段:智能] OR "腹针"[常用字段:智能] OR "干针"[常用字段:智能]

3 1 OR 2

4 "泌乳障碍"[加权:扩展] OR "母乳喂养"[加权:扩展]

5 "缺乳"[常用字段:智能] OR "乳少"[常用字段:智能] OR "少乳"[常用字段:智能] OR "乳汁"[常用字段:智能] OR "泌乳"[常用字段:智能] OR "母乳"[常用字段:智能] OR "哺乳"[常用字段:智能]

6 4 OR 5

7 "随机对照试验" [加权:扩展] OR "临床试验"[加权:扩展]

8 "随机"[常用字段:智能] OR "临床研究"[常用字段:智能] OR "临床试验"[常用字段:智能] OR "RCT"[常用字段:智能]

9 7 OR 8

10 3 AND 6 AND 9

WanFang:

(主题:("针刺" OR "针灸" OR "体针" OR "手针" OR "电针" OR "头针" OR "耳针" OR "腹针" OR "干针")) and (主题:("缺乳" OR "乳少" OR "少乳" OR "乳汁" OR "泌乳" OR "母乳" OR "哺乳")) and (主题:("随机" OR "临床研究" OR "临床试验" OR "RCT"))

VIP:

M=(针刺 OR 针灸 OR 体针 OR 手针 OR 电针 OR 头针 OR 耳针 OR 腹针 OR 干针) and M=(缺乳 OR 乳少 OR 少乳 OR 乳汁 OR 泌乳 OR 母乳 OR 哺乳) and R=(随机 OR 临床研究 OR 临床试验 OR RCT)
