# Supplementary material for: Efficacy and safety of acupuncture for postpartum hypogalactia: A systematic review and meta-analysis of randomized controlled trials
Source: PLoS One. 2024 Jun 6;19(6):e0303948. doi: 10.1371/journal.pone.0303948 (PMC11156417; doi:10.1371/journal.pone.0303948)
Supplement: Attachment — Submitted filename: Response to Reviewers.docx [file pone.0303948.s008.docx]

Dear Editors and Reviewers:

We sincerely appreciate for the editors and reviewers’ comments concerning our manuscript entitled “Efficacy and safety of acupuncture for postpartum hypogalactia: A systematic review and meta-analysis of randomized controlled trials”. Those comments are all valuable and very helpful for revising and improving our paper, as well as the important guiding significance to our review. We have studied comments carefully and have made correction which we hope meet PLOS ONE’s publication criteria. Revised portions are marked red in the paper. The main corrections in the paper and the responds to the editor and reviewers’ comments are as following:

**Editor’s Comments:**

MD. Benjamin Jun Jie Seng:

1. Please ensure that your manuscript meets PLOS ONE's style requirements, including those for file naming. The PLOS ONE style templates can be found at.

**Response:** Many thanks for your kindly comment. We have carefully read the PLOS ONE style templates you provided, and revised our manuscript according to the requirements, including file naming, title, authors, affiliations and main body.

2. We note that the grant information you provided in the ‘Funding Information’ and ‘Financial Disclosure’ sections do not match.

When you resubmit, please ensure that you provide the correct grant numbers for the awards you received for your study in the ‘Funding Information’ section.

**Response:** Thanks for your significant comment. We have checked and revised the grant information in the ‘Funding Information’ section. This study was financially supported by the “Central Financial Transfer Payment to Local Projects in 2022 of National Administration of Traditional Chinese Medicine”, which does not have a specific grant number. Fanrong Liang was the study funder.

3. PLOS requires an ORCID iD for the corresponding author in Editorial Manager on papers submitted after December 6th, 2016. Please ensure that you have an ORCID iD and that it is validated in Editorial Manager. To do this, go to ‘Update my Information’ (in the upper left-hand corner of the main menu), and click on the Fetch/Validate link next to the ORCID field. This will take you to the ORCID site and allow you to create a new iD or authenticate a pre-existing iD in Editorial Manager. Please see the following video for instructions on linking an ORCID iD to your Editorial Manager account: https://www.youtube.com/watch?v=_xcclfuvtxQ

**Response:** Thanks for this important guidance. I have seen the instructed video and linked an ORCID iD in my Editorial Manager account.

**Additional Editor Comments:**

1. Methods

- The search period is slightly outdated and nearly done 1 year from date of submission

-> It will benefit from an update

**Response:** Thanks for your significant comment. We have performed an updated search on October 19, 2023. 1 more RCT was included in this systematic review and relevant results have been updated.

2. Table 2

- Standardize the reporting and number of significant figures reported for the results

- Suggest to put the actual treatment instituted in the control group

-> This has implications on the suitability of meta-analyses as if there are drastically different control measures implemented, the pooling of the results may not be appropriate.

-> the authors may need to check on this

**Response:** Thanks a lot for your significant suggestion. We have standardized the reporting and number of significant figures reported for the results. Based on currently available original data, sham acupuncture, conventional treatment (breast sucking, postpartum routine care, breastfeeding education) and Chinese herb were included as control in this systematic review. Studies used interventions such as drugs, galactagogues, massage, or any other complementary therapy in control group were not included. The pooled data from included RCTs are divided into comparators including acupuncture + control vs. control and acupuncture vs. control. We have described the detailed control measures and treatment in the Methods and Results section.

3. Discussion

- Brief discussion should be made with regards to role of acupuncture in postpartum hypogalactia as to how it should be used in current practice

- Are there any patients whom acupuncture should not be used?

**Response:** Thank you for this precious suggestion. Our findings may provide guidance for the clinical treatment of PH. We have discussed how to use acupuncture to treat postpartum hypogalactia in practice from the perspective of better combination modality, treatment duration, acupoint selection, and traditional Chinese medicine (TCM) syndrome differentiation. Concerning safety, minor adverse effects of acupuncture were recorded in three studies, and these responses returned to normal after treatment. Therefore, acupuncture could be considered a relatively safe intervention to improve milk supply for women with PH. Considering that few participants in the acupuncture group experienced skin itching and negative sensations, it may not suitable for those who fear of needling or allergic to metal. We have added the in the Discussion section.

**Reviewer #1:**

1. Were there any attempts made to look for unpublished data which could have affected the evaluation of the meta-analysis?

**Response:** Thanks a lot for your important comment. We have retrieved clinical registry platform for unpublished data, and no available unpublished data was found.

2. Table 2: what is TCM syndrome differentiation? Table 2 is also difficult to read with many acronyms and many different outcomes being reviewed. Are there certain standardized or commonly used parameters we can focus on instead of evaluating such a wide range of efficacy and safety parameters?

**Response:** Thanks for your significant suggestion. Treatment based on TCM syndrome differentiation is the core principle of TCM; that is means taking the individual as the study’s starting point and analyzing the pathophysiological characteristics, nature of lesions and development trend according to their clinical signs and symptoms, so as to develop the corresponding TCM treatments. Many studies have demonstrated that TCM syndrome differentiation have value in predicting prognosis and improving intervention effects in various diseases. In this systematic review, thirteen of the included studies included participants based on the TCM syndrome differentiation, including deficiency of Qi and blood syndrome; liver-Qi stagnation syndrome; deficiency of spleen-Qi syndrome; phlegm dampness and blood stasis syndrome; Qi deficiency and blood stasis syndrome. We have added information regarding Traditional Chinese medicine (TCM) syndrome differentiation in study characteristic.

We have revised the Table 2. It was complicated and hard to understand. For outcome measures, we retained some standardized and commonly used parameters, including serum prolactin level, milk secretion volume, mammary fullness degree, total effective rate, exclusive breastfeeding rate, and adverse events.

3. Table 5: Inappetence > can be replaced with "Loss of appetite"

"Dizzy" > can be replaced with "Dizziness"

"Breast distending" > can be replaced with "breast distension"

Please complete what "3 cases of gastrointestinal" means. Do you mean "gastrointestinal discomfort"?

**Response:** Thank you very much for your careful review. We have modified the above inappropriate words in the Table 3.

**Reviewer #2:**

Interesting study. Some issues need clarifications.

1. Write the full details of search strategy with the keywords used and results of each data base individually

**Response:** Many thanks for your significant comment. We have added the detailed search strategies of each database in the S1 file.

2. Study characteristics add the inclusion and exclusion criteria of participants

**Response:** Thanks for your important comment. We have added information regarding the inclusion and exclusion criteria of participants in the Study characteristics section. A total of eleven of the included studies described the inclusion and exclusion criteria of participants in detail.

3. Add the registration details of each study prospective or retrospective or not registered

**Response:** Thanks for your kindly recommendation. All the included studies in this systematic review have not registered in the clinical registry platform in advance. We have added related information in the Results and Discussion section.

4. Subgroup analysis according to time frequency type and duration of intervention should be added

**Response:** Thanks a lot for your advice. We have added subgroup analyses according to duration of intervention, the results have been presented in the Results section. But subgroup analysis based on time frequency type cannot be performed, because all studies except 1 were treated once a day.
