# Supplementary material for: Efficacy and safety of acupuncture for postpartum hypogalactia: A systematic review and meta-analysis of randomized controlled trials
Source: PLoS One. 2024 Jun 6;19(6):e0303948. doi: 10.1371/journal.pone.0303948 (PMC11156417; doi:10.1371/journal.pone.0303948)
Supplement: Attachment — Submitted filename: Response_to_Reviewers.docx [file pone.0303948.s009.docx]

Dear Editors and Reviewers:

We sincerely appreciate the editors and reviewers’ comments concerning our manuscript entitled “Efficacy and safety of acupuncture for postpartum hypogalactia: A systematic review and meta-analysis of randomized controlled trials”. Those comments are all valuable and very helpful for revising and improving our paper, as well as the important guiding significance to our review. We have studied the comments carefully and have made corrections to meet PLOS ONE’s publication criteria. The revised portions are marked red in the paper. The main corrections in the paper and the response to the editor and reviewers’ comments are as follow:

**Reviewers' comments:**

Reviewer's Responses to Questions

Comments to the Author

1. If the authors have adequately addressed your comments raised in a previous round of review and you feel that this manuscript is now acceptable for publication, you may indicate that here to bypass the “Comments to the Author” section, enter your conflict of interest statement in the “Confidential to Editor” section, and submit your "Accept" recommendation.

Reviewer #1: All comments have been addressed

**Response:** We greatly appreciate your review and support for the study.

Reviewer #3: (No Response)

**Response:** We are grateful for your significant comments. We have carefully read and addressed the manuscript in this round according to your comments.

2. Is the manuscript technically sound, and do the data support the conclusions?

The manuscript must describe a technically sound piece of scientific research with data that supports the conclusions. Experiments must have been conducted rigorously, with appropriate controls, replication, and sample sizes. The conclusions must be drawn appropriately based on the data presented.

Reviewer #1: Yes

**Response:** Thank you very much for your agreement.

Reviewer #3: Partly

**Response:** We extend our many thanks for your review. We have further modified the results of the meta-analysis and presented it.

3. Has the statistical analysis been performed appropriately and rigorously?

Reviewer #1: Yes

**Response:** Thank you very much for this approval.

Reviewer #3: No

**Response:** We sincerely thank you for your comments. We have revised the statistical analysis in a more appropriate manner.

4. Have the authors made all data underlying the findings in their manuscript fully available?

The PLOS Data policy requires authors to make all data underlying the findings described in their manuscript fully available without restriction, with rare exception (please refer to the Data Availability Statement in the manuscript PDF file). The data should be provided as part of the manuscript or its supporting information, or deposited to a public repository. For example, in addition to summary statistics, the data points behind means, medians and variance measures should be available. If there are restrictions on publicly sharing data—e.g. participant privacy or use of data from a third party—those must be specified.

Reviewer #1: Yes

**Response:** Many thanks for your consent.

Reviewer #3: Yes

**Response:** Many thanks for your consent.

5. Is the manuscript presented in an intelligible fashion and written in standard English?

PLOS ONE does not copyedit accepted manuscripts, so the language in submitted articles must be clear, correct, and unambiguous. Any typographical or grammatical errors should be corrected at revision, so please note any specific errors here.

Reviewer #1: Yes

**Response:** Thank you very much for this ratification.

Reviewer #3: Yes

**Response:** Thank you very much for this ratification.

**6. Review Comments to the Author**

Please use the space provided to explain your answers to the questions above. You may also include additional comments for the author, including concerns about dual publication, research ethics, or publication ethics. (Please upload your review as an attachment if it exceeds 20,000 characters)

Reviewer #1: No further comments, my comments have been addressed.

This topic is of interest as PH is a prevalent issue among breastfeeding mothers. It is useful to know the utility of acupuncture in this area as this is something that is not commonly considered by layman as treatment for PH.

**Response:** Thank you very much for your acceptance and approval.

Reviewer #3: 1. The studies differ significantly in terms of interventions and control arms. Authors should present data as following comparators (MA+CT vs CT, MA+CH vs CH, MA vs CH and MA vs CT, etc) in one forest plot as subgroup or refrain from conducting meta-analysis and present finding as systematic review only. This will reduce the number of figures and give better clarity to manuscript.

**Response:** Thank you for your comments. We have modified the meta-analysis. For each outcome, different comparators (MA+CT vs CT, MA+CH vs CH, MA vs CH and MA vs CT, etc) were presented as subgroups in one forest plot. The number of figures has been reduced, and all results of meta-analysis have been corrected.

2. Other concern is inclusion of selection of studies that did not report the selection criteria (8 studies) and treatment duration (3 studies) besides high concern in randomization. The findings of meta-analysis depend on kind of included studies. Overall, poor quality studies are included in the meta-analysis.

**Response:** Thank you for your comments. The studies that did not report the selection criteria or treatment duration have been rated as “some concern” in the domain of selection of reported results. We conducted a systematic search through databases from their establishment to October 2023; unfortunately, this led to studies of poor quality being included in this systematic review and meta-analysis, which is a significant limitation of this study. We have described this limitation and put forward relevant recommendation for future research in the Discussion section.

3. I do not agree with GRADE evidence quality of outcome. Authors has considered publication bias “undetected” for the outcomes “PRL, MSV, MFD and EBR”. However, authors have made no attempt to detect publication bias for these outcomes due to less than 10 studies. I do not agree with this assessment. Funnel plots are most likely asymmetrical for these outcomes. Publication should be “suspected” for these outcomes. The quality of evidence must be downgraded to “Very low/critically low”.

**Response:** Many thanks for your important comments. We have revised the assessment of publication bias into “strongly suspected” for the outcomes of serum prolactin level, milk secretion volume, mammary fullness degree, and exclusive breastfeeding rate, and the quality of evidence has been downgraded to “critically low”.
