# Supplementary material for: Efficacy and safety of acupuncture for postpartum hypogalactia: A systematic review and meta-analysis of randomized controlled trials
Source: PLoS One. 2024 Jun 6;19(6):e0303948. doi: 10.1371/journal.pone.0303948 (PMC11156417; doi:10.1371/journal.pone.0303948)
Supplement: Attachment — Submitted filename: Response to Reviewers and Editors.docx [file pone.0303948.s010.docx]

Dear Editors:

We sincerely appreciate for the journal requirements and editor's comments concerning our manuscript entitled “Efficacy and safety of acupuncture for postpartum hypogalactia: A systematic review and meta-analysis of randomized controlled trials”. Those comments are all valuable and very helpful for revising and improving our paper, as well as the important guiding significance to our review. We have studied comments carefully and have made correction which we hope meet PLOS ONE’s publication criteria. Revised portions are marked red in the paper. The main corrections in the paper and the responds to the journal requirements and editor’s comments are as following:

**Journal Requirements:**

Please review your reference list to ensure that it is complete and correct. If you have cited papers that have been retracted, please include the rationale for doing so in the manuscript text, or remove these references and replace them with relevant current references. Any changes to the reference list should be mentioned in the rebuttal letter that accompanies your revised manuscript. If you need to cite a retracted article, indicate the article’s retracted status in the References list and also include a citation and full reference for the retraction notice.

**Response:** Thank you for bringing this to our attention. We have thoroughly reviewed the reference list to ensure its completeness and accuracy. We have also verified that no retracted papers have been cited in the manuscript. Your reminder is greatly appreciated, and we are committed to maintaining the highest standards of integrity in our research.

**Additional Editor Comments:**

Dear authors,

The revision are mostly satisfactory.

Minor comments

- Multiple grammatical errors still remains

- Dear authors,

Abstract

- The databases used for the review should be stipulated

**Response:** Thank you for your positive feedback and support for our study. We have taken note of your suggestions and have worked to improve the language, grammar, and overall clarity of the manuscript. We have also included the databases used in the review in the Abstract as recommended. We strive to ensure that the manuscript is error-free and meets the highest standards of quality. Thank you for your valuable input.
